# Evaluation of Drying Process on the Composition of Black Pepper Ethanolic Extract by High Performance Liquid Chromatography With Diode Array Detector

**Published:** 2012-10-07

**Authors:** Foroogh Namjoyan, Hoda Hejazi, Zahra Ramezani

**Affiliations:** 1 Marine Research Center, Pharmacognosy Departments, Faculty of Pharmacy, Jundishapur University of Medical Sciences, Ahvaz, IR Iran; 2 Nanotechnology Research Center, Faculty of Pharmacy, Jundishapur University of Medical Sciences, Ahvaz, IR Iran; 3 Faculty of Pharmacy, Jundishapur University of Medical Sciences, Ahvaz, IR Iran

**Keywords:** Freeze Drying, Black Pepper, Piperine

## Abstract

**Background:**

Black pepper (Piper nigrum) is one of the well-known spices extensively used worldwide especially in India, and Southeast Asia. The presence of alkaloids in the pepper, namely, piperine and its three stereoisomers, isopiperine, chavicine and isochavicine are well noticed.

**Objectives:**

The current study evaluated the effect of lyophilization and oven drying on the stability and decomposition of constituents of black pepper ethanolic extract.

**Materials and Methods:**

In the current study ethanolic extract of black pepper obtained by maceration method was dried using two methods. The effect of freeze and oven drying on the chemical composition of the extract especially piperine and its three isomers were evaluated by HPLC analysis of the ethanolic extract before and after drying processes using diode array detector. The UV Vis spectra of the peaks at piperine retention time before and after each drying methods indicated maximum absorbance at 341.2 nm corresponding to standard piperine.

**Results:**

The results indicated a decrease in intensity of the chromatogram peaks at approximately all retention times after freeze drying, indicating a few percent loss of piperine and its isomers upon lyophilization. Two impurity peaks were completely removed from the extract.

**Conclusions:**

In oven dried samples two of the piperine stereoisomers were completely removed from the extract and the intensity of piperine peak was increased.

## 1. Background 

**Figure 1:**
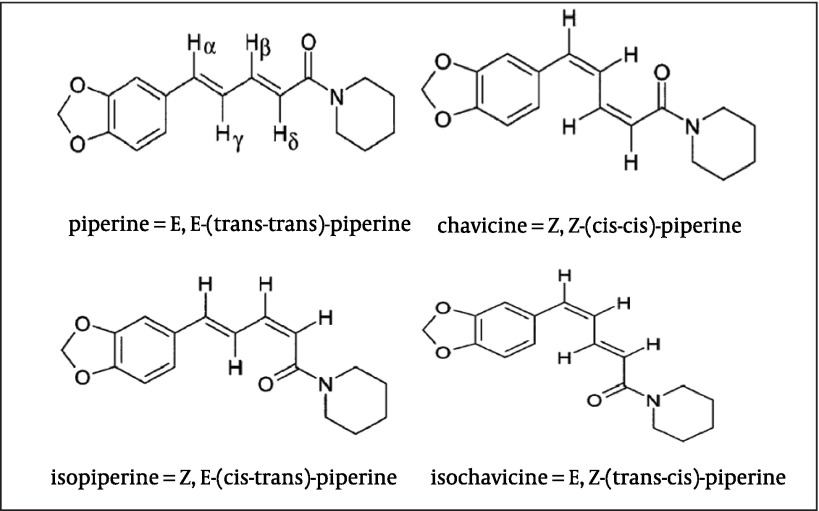
Chemical Structure of Different Piperine Isomers

Black pepper (Piper nigrum) is one of the well known spices extensively used worldwide especially in India and Southeast Asia. The presence of alkaloids in pepper, namely, piperine and its three stereoisomers, isopiperine, chavicine and isochavicine are well noticed (1). Their chemical structures are presented in Figure 1. The main constituent of pepper is piperine. Piperine is responsible for many pharmacological activities of the black pepper. It is used for its anti-inflamatory, anti-oxidant, antibacterial, antitumor, and heptaprotective activities (2). In order to control the side effects of black pepper used orally, piperine is extracted and purified from black pepper to present specified herbal products (3). Piperine is extracted from black pepper by different extraction methods such as super critical fluid extraction (SFE), Soxhelet and maceration using 95% ethanol and methanol or chloroform as solvent, and then purified by different procedures (1, 3-5).

Some botanical samples are freeze dried before their use as food, spices, or in research studies. Although some belive this method preserves the quality of these plants, little systematic research is available to prove the claim. Freeze drying of the samples has diverse effect on different constituents of the plants. Volatile profile of the plants may vary on freeze drying. Supposedly it may increase or degrade the volatile contents compared to other drying methods such as hot and ambient air and oven drying methods (6). Freeze drying preserved more volatile aroma of dill (Anethum graveolens) compared to hot air drying (7). Another report indicated that the thymole contents of the plant increased after freeze drying (8). Most researches indicate thereduction or degradation of the volatiles after freeze drying (9-11). Regarding the other components, in most plants freeze drying keeps their phenolic and antioxidant contents (12-15). As for carotenoids, in some cases freeze drying acts better than other drying methods (16). Freeze drying of Echinacea purpurea causes more retention of cichoric acid, a moisture sensitive acid, than air drying (17, 18). Freeze drying affects different plants and different constituents in a variety of ways. So it is still required to check its effect on components of individual plants.

## 2. Objectives 

The current study evaluated the effect of lyophilization and oven drying on the stability and decomposition of constituents of black pepper ethanolic extract.

## 3. Materials and Methds

### 3.1. Reagents and Materials

HPLC grade solvents such as acetonithryl, ethanol, methanol and Phosphoric acid and phosphaste salts were obtained from Merck (Germany). Standard Piperine was obtained from sigma-Aldrich with 98% percent purity. Black pepper seeds were obtained from local herbal shop. Chromofil HPLC filter 0.2 µm, 25mm (Germany) were used to filter solutions prior to injection to HPLC. Double distilled water was used throughout the current study.

### 3.2. Instrumentations

Samples were freeze dried by an Operon FDCF instruments (Korea). HPLC measurements were carried out on a Waters 600 instrument (USA) equipped with on line degasser, column oven, and photo diod array detector (DAD). Separation was done on a C18 Waters (250×4.6 mm) 5µm ODS2 column. Mobile phase consisted of pH 4.5 phosphate buffer and acetonitril (1:1). Detector was set to collect the spectral information of each retention time in the range of 200 to 400nm and record the chromatograms at 340 nm.

### 3.3. Piperine Extraction and Lyophilization

For extraction 45 mg of black pepper powder and 60 mL of 95% ethanol were mixed completely. The suspension was stored overnight and then filtered using vacuum filtration. Filtrate was divided into three portions of 20 mL. Twenty five micro liter of the first portion was injected to HPLC immediately. The second portion was freeze dried and the third portion was oven dried. Remaining yellow residue of the second and third portions were redissolved in 20 mL of ethanol %95 and 25µl of this solution was injected to HPLC and its chromatograms were recorded and processed.

## 4. Results 

**Figure 2:**
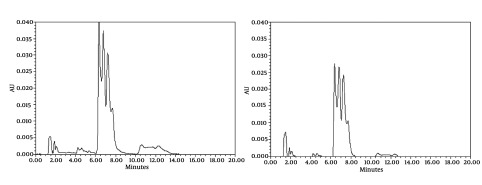
HPLC Analysis of Black Pepper Extract A) Before B) After Lyophilization (Chromatographic Conditions)

**Figure 3:**
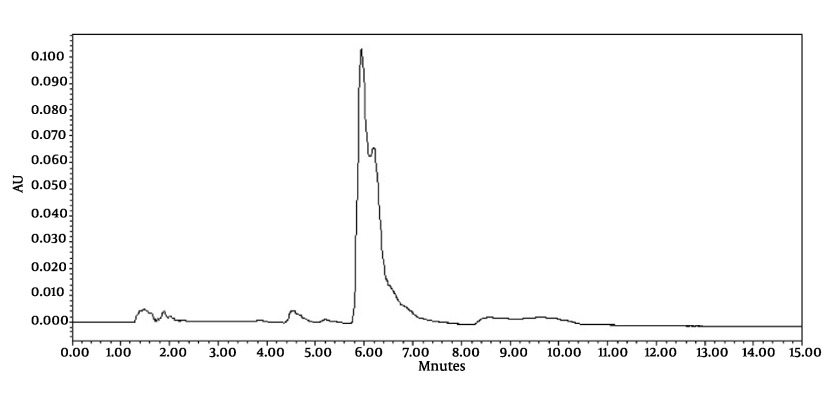
Chromatogram of Oven Dried Samples (Chromatographic Conditions)

Chromatograms of freshly prepared and freeze dried extract of black pepper are presented in Figure 2. Figure 3 indicates oven dried sample chromatogram. By comparing these three chromatograms it can be concluded that freeze drying decreases the amount of piperine and its three streoisomers. Two impurity peaks at retention times 5.4 and 12 min were completely removed from the extract. These two peaks are not desired and usually more purification is required to remove them from ethanolic extract. Table 1 indicates the decrease in each component of the extract after applying the drying methods. 43%reduction in piperine content of the extract (R_T_ = 6.4) was observed in freeze dried samples while oven drying increased the peak intensity and purity of the piperine as can be concluded by comparing the Figure 3 and Figure 2.

**Figure 4:**
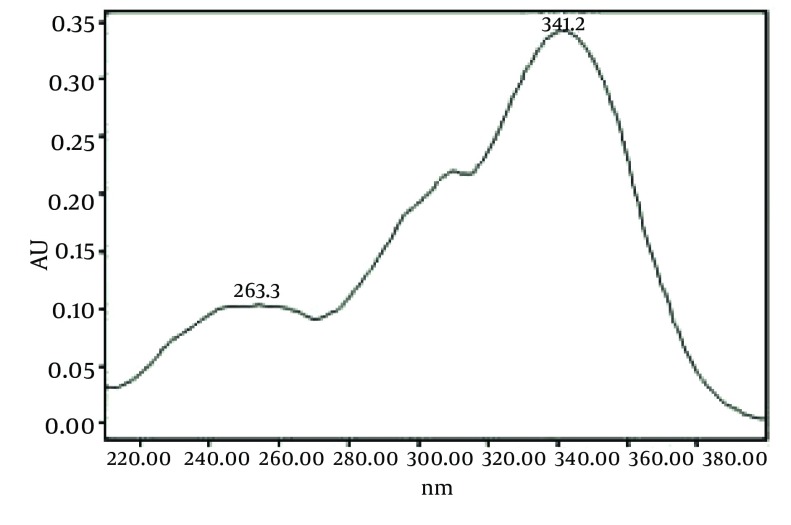
UV Vis Spectra of Piperine Standard Extracted From HPLC-DAD Chromatogram at R_t_ 6.4 min

**Figure 5:**
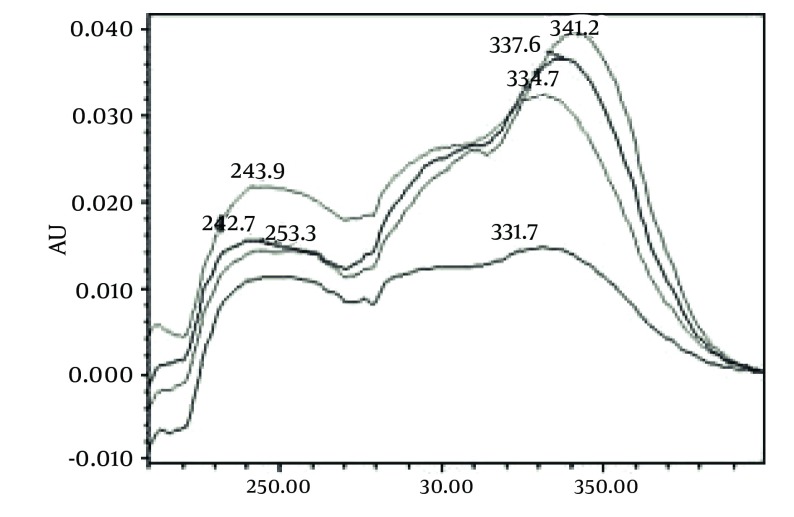
UV/Vis Spectra of Piperine and Other its Three Isomers Extracted From HPLC-DAD Chromatogram of Non Freeze Dried Samples at R_t_ 6 to 8 min

**Figure 6:**
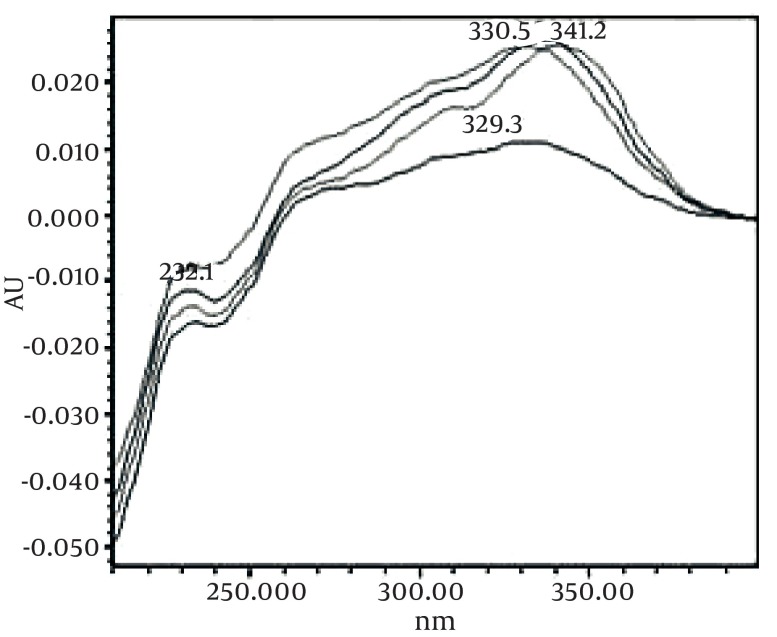
UV/Vis Spectra of Piperine and Other it’s Three Isomers Extracted From HPLC-DAD Chromatogram of Freeze Dried Samples at R_t_ 6 to 8 min.

Figure 4 is the standard piperine UV/Vis spectra extracted from its correspounding chromatogram at 6.4 min collected in the previously described chromatographic conditions. UV/Vis spectra of piperine and three stereoisomers at corresponding retention times of the extract before and after lyophilization were compared with those of standard. These spectrums are presented in Figures 5 and 6. As it is obvious from the figures piperine in each case shows a maximum at 341.2 nm corresponding to maximum wavelength of piperine. Other stereoisomers appeared at 6.8, 7.2 and 7.7 minutes as compared with the literature results of HPLC analysis of the mixtures (19).

**Table 1:** Chromatograms Retention Times and the Percent Decrease in Each Compound After Drying

	Reduction, %	
**R_T_**	**Oven Dried**	**Freeze Dried**
5.4	28.31	100
6.4	0.0	33.0
6.8	0.0	25.5
7.2	100	18
7.7	100	17.8
10.6	0.5	47.5
12.3	22.94	100

## 5. Discussion 

**Figure 7:**
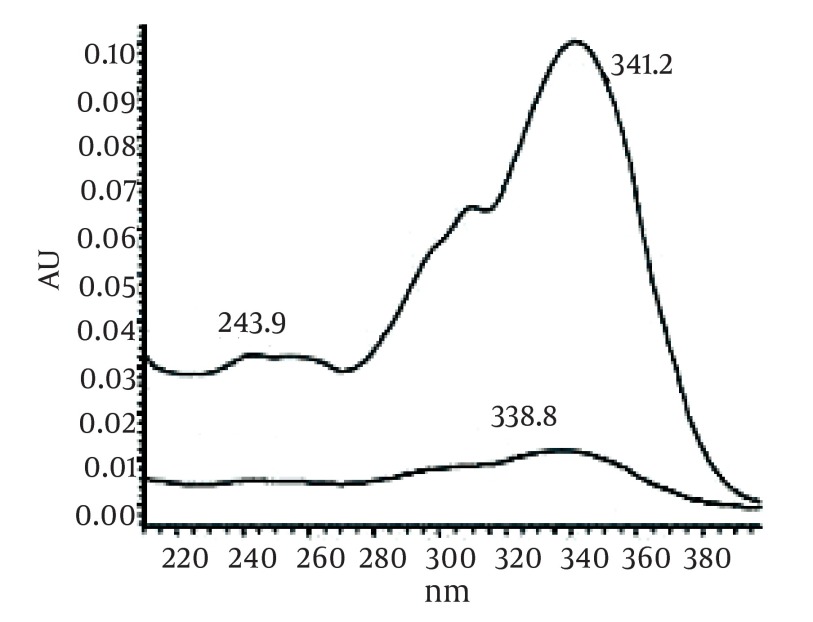
UV/Vis Spectra of Oven Dried Samples at Rt = 6.4 and 6.8

By comparing these two UV/Vis spectra, it can be concluded that no decomposition occurred for piperine and their three streoisomers but 17-33% reduction in concentration occurred as indicated in Table 1. UV/Vis spectra of the oven dried spectra at piperine and its stereoisomer’s retention time is presented in Figure 7. As it can be observed by comparing the chromatogram and the extracted UV/Vis spectra, two of streoisomers are completely eliminated from the oven dried samples. It seems that oven drying causes conversion of isomers to piperine since about 30-50% increase in the piperine intensity is observed. As the results indicate the lyophilization eliminates some impurities in the ethanolic extract but loss of piperine contents is also observed to some extent. Instead, oven drying increases the piperine content of the extract and eliminates two of the streoisomers. in order to find the possible mechanism more detailed study is required. It can also be concluded that impurities are higher in oven dried samples.
